# Identification of Interleukin‐1β in Whole Blood as a Candidate Biomarker for Alcohol Use Disorder Risk Based on AUDIT Scores

**DOI:** 10.1111/adb.70088

**Published:** 2025-09-16

**Authors:** Irina Balan, Alejandro G. Lopez, Thomas Gilmore, Michael Bremmer, Todd K. O'Buckley, Kai Xia, Christian S. Hendershot, A. Leslie Morrow

**Affiliations:** ^1^ Bowles Center for Alcohol Studies, School of Medicine The University of North Carolina at Chapel Hill Chapel Hill North Carolina USA; ^2^ Department of Psychiatry, School of Medicine The University of North Carolina at Chapel Hill Chapel Hill North Carolina USA; ^3^ Department of Biochemistry and Biophysics University of North Carolina at Chapel Hill Chapel Hill North Carolina USA; ^4^ Department of Psychology The University of North Carolina at Chapel Hill Chapel Hill North Carolina USA; ^5^ Department of Pharmacology, School of Medicine The University of North Carolina at Chapel Hill Chapel Hill North Carolina USA

**Keywords:** AUD risk, cytokines, IL‐1β, immune dysregulation, inflammation, luminex multiplex assay, machine learning, principal component analysis, random forest, ROC analysis

## Abstract

Alcohol use disorder (AUD) is associated with chronic inflammation and immune dysregulation, yet no validated immune‐based markers exist to support assessment or monitoring. This study identifies interleukin‐1 beta (IL‐1β) in whole blood as a promising candidate biomarker of AUD risk, based on Alcohol Use Disorders Identification Test (AUDIT) scores. Twenty‐eight non–treatment‐seeking adults, with AUDIT scores between 2 and 22, provided whole blood samples. We aimed to identify biomarkers that signal immune changes associated with early AUDIT score risk, where interventions may be most effective. Luminex multiplex immunoassays quantified 14 immune‐related mediators in combined cell lysates and supernatants. IL‐1β, IL‐18, IL‐7 and CCL11 were significantly elevated in individuals with higher AUDIT scores. IL‐1β showed the largest effect size (Cohen's *d*) and was the most consistent predictor of both AUDIT and AUDIT‐Consumption (AUDIT‐C) scores across random forest and linear regression analyses. Moderated multiple regression (MMR) confirmed that IL‐1β predicted both scores independent of other immune mediators. Receiver operating characteristic (ROC) analyses demonstrated discriminative potential, with IL‐1β achieving an AUC of 0.81 (good discrimination) for AUDIT ≥ 6 (true positive rate [TPR] = 0.71; false positive rate [FPR] = 0.14) and an AUC of 0.94 (excellent discrimination) for AUDIT‐C thresholds (TPR = 0.80; FPR = 0.00). Principal component analysis (PCA) revealed greater immune variability in the high‐risk group, particularly among proinflammatory mediators, suggesting immune dysregulation. This study demonstrates the utility of integrating whole blood immune profiling with high‐sensitivity multiplex immunoassays, and applying both traditional statistical methods and machine learning to explore potential biomarkers for AUD risk. IL‐1β is a statistically robust and clinically relevant candidate biomarker of AUD risk assessed by AUDIT scores. These findings require replication in larger, independent samples to determine their translational potential in addiction medicine.

## Graphical abstract

IL‐1β in whole blood is identified as a key biomarker of AUD risk based on AUDIT scores. In 28 non‐treatment‐seeking adults, multiplex immunoassays combined with traditional statistics and machine learning ranked IL‐1β as the top predictor, showing good discrimination (AUC=0.81) and elevated levels in the group with AUDIT ≥ 6. PCA revealed immune heterogeneity in this group, highlighting immune dysregulation even at moderate‐risk levels. This integrative whole blood approach supports IL‐1β as a promising biomarker for early AUD detection.

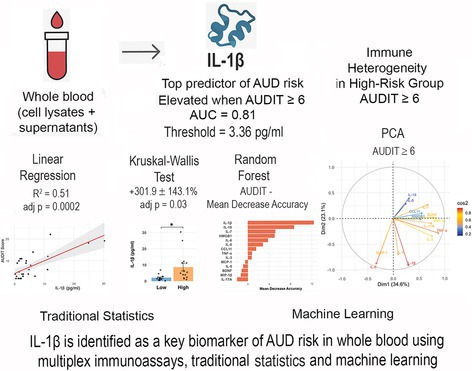

## 1 Introduction

Alcohol use disorder (AUD) is a chronic, relapsing condition that affects millions globally and contributes to significant physical, mental and social harm [1, 2]. AUD is widely underdiagnosed and untreated. The availability of reliable biological markers might help to improve rates of diagnosis and monitoring. While validated tools like the Alcohol Use Disorders Identification Test (AUDIT) and its shorter version, AUDIT‐C, are widely used to screen for alcohol‐related problems [3, 4], they are prone to recall bias and underreporting. Identifying objective, biologically based markers of AUD could improve diagnostic accuracy and support more personalized intervention strategies.

Growing evidence links AUD to chronic inflammation and immune dysregulation across multiple organ systems, including the liver, gut and brain. Prolonged alcohol exposure activates peripheral and central immune responses, contributing to blood–brain barrier disruption, neuroinflammation and altered cytokine signalling in brain regions involved in mood, cognition and addiction [1, 5, 6, 7, 8, 9, 10, 11, 12, 13, 14, 15]. Systemic inflammation is also associated with organ damage, increased infection risk and poorer treatment outcomes in individuals with AUD [12, 14]. Craving in AUD has also been significantly associated with gut dysregulation and candidate proinflammatory markers such as IL‐1β and TNF‐α, further linking immune activity with clinical symptomatology [16]. These findings underscore the need for immune‐based approaches to better characterize the biological impact of AUD and identify clinically relevant biomarkers [17].

The analysis of cytokine and chemokine levels in clinical blood specimens is increasingly recognized as a valuable approach for understanding immune alterations in AUD [17, 18, 19, 20]. These immune signalling molecules play central roles in regulating inflammation and intercellular communication, and their dysregulation may reflect underlying neuroimmune disturbances associated with chronic alcohol exposure. Quantifying their expression in blood could provide insight into the immunopathology of AUD and support the development of objective biomarkers for diagnosis and monitoring. Experimental evidence further suggests that immune challenges, such as endotoxin administration, can modulate craving in individuals with AUD, underscoring the complex and potentially causal role of immune activation in AUD symptomatology [21]. However, few studies have examined immune biomarkers alongside validated AUD screening tools [15, 22, 23, 24], and none, to our knowledge, have assessed them in the combination of supernatants and cell lysates from whole blood—the most comprehensive source of immune signals. Additionally, the predictive value of biomarkers using machine learning remains largely unexplored.

In this study, we analysed whole blood samples from 28 non–treatment‐seeking adult participants representing a range of alcohol use severity, as assessed by AUDIT and AUDIT‐C scores. We quantified 14 immune‐associated mediators using Luminex multiplex assays on combined supernatant and lysate fractions and applied linear regression, random forest modelling, moderated multiple regression (MMR), receiver operating characteristic (ROC) analysis and principal component analysis (PCA) to evaluate associations with AUDIT and AUDIT‐C scores, predictive value and immune‐related pattern differences between high‐ and low‐risk alcohol consumption groups.

## 2 Participants and Methods

### 2.1 Participants and Recruitment

Adult participants (*N* = 40, female = 23, male = 17) were recruited from the University of North Carolina (UNC) at Chapel Hill, NC, USA, and the surrounding community. Social media advertisements, community ads and UNC's research recruitment website were utilized with language targeting non–treatment‐seeking adults with varying levels of alcohol consumption. Interested participants responded to advertisements and completed a phone screening to determine initial eligibility. Potentially eligible participants then completed a video screening appointment that included electronic informed consent and additional screening questions. All procedures were approved by the UNC Institutional Review Board.

Inclusion criteria included current (past month) alcohol consumption, fluency in English and age 21–45. Participants in the high‐risk group were required to have consumed, in the past 30 days, at least five drinks in a day or 15 drinks per week for men, or at least four drinks in a day or eight drinks per week for women. Participants in the low‐risk group were required to endorse fewer than six drinks per week and no heavy drinking episodes in the past 30 days, no lifetime history of moderate or severe AUD, and no significant reductions in drinking (50% reduction or more in drinks per week) over the past year. Exclusion criteria included past‐year criteria for another substance use disorder (SUD; with exception of mild cannabis use disorder), currently receiving treatment or planning to seek treatment for alcohol use concerns or a lifetime diagnosis of severe mental illness. Participants were also excluded if they endorsed autoimmune or inflammatory diseases (e.g., Crohn's disease and inflammatory bowel disease), screened positive for current depression (based on a PHQ‐9 score of 10 or greater) or were receiving current treatment for depression (given that depression may be associated with inflammation and AUD diagnosis). Finally, exclusion criteria included weekly or greater use of cigarettes or electronic nicotine delivery systems (ENDS), fear of needles or history of adverse reactions to blood draws and pregnancy or nursing. Eligible participants were scheduled for a single laboratory visit consisting of a blood draw and an assessment interview.

### 2.2 Assessment Instruments

The AUDIT is a 10‐item screening measure to assess the severity of alcohol consumption/hazardous drinking [25]. The AUDIT includes questions that evaluate alcohol consumption, alcohol‐related problems and dependence symptoms. A total score is derived from a sum of all items. Additionally, a consumption score (AUDIT‐C) is derived from the sum of the first three items. Total scores range from 0 to 40, with higher scores indicating greater risk for alcohol dependence (0–7: low risk; 8–15: at‐risk drinking; 16–19: hazardous or harmful drinking; 20+ possible alcohol dependence) [26]. AUDIT cutoffs examined in the present study included the conventional cutoff of 8+ and a more conservative cutoff of 6+ (see analysis plan). Additionally, AUDIT‐C scores of 3 for women and 4 for men (the conventional cutoffs for indexing at‐risk drinking) were evaluated [27]. The structured clinical interview for DSM‐5 disorders (SCID‐5; Module E) [28] was administered to assess for AUD and SUD symptoms. However, because the study was focused on identifying immune‐related biomarkers associated with alcohol use severity, we did not perform formal DSM‐5 diagnostic classification. We shifted our recruitment strategy to focus on AUDIT and AUDIT‐C scores after observing stronger biomarker signals relative to DSM‐based AUD diagnoses early in the study (data not shown). Thus, participants were stratified based on alcohol use severity using AUDIT and AUDIT‐C scores, which are validated screening tools [3, 4, 25, 26, 27]. Additionally, a 90‐day Timeline Followback (TLFB) interview was administered to characterize recent drinking history [29].

Alcohol craving was measured using the Penn Alcohol Craving Scale (PACS), a validated five‐item self‐report questionnaire that measures the frequency, intensity and duration of alcohol craving over the past week. Each item is rated on a 7‐point Likert scale (0–6), yielding a total score ranging from 0 to 30, with higher scores indicating greater craving. The PACS was administered electronically using REDCap during the laboratory session, along with other clinical assessments [30]. While PACS scores were used for descriptive group comparisons (see Table 1), they were not included in immune‐related biomarker analyses.

**TABLE 1:** Demographic, clinical and drinking pattern differences between low‐ and high‐risk alcohol consumption groups.

	Mean(SD)		Total
	Low drinking	High drinking	
*N*, no. (male/female)	7/7	7/7	14/14
Age, years	27.1(3.3)	25.9(4.3)	26.5(3.8)
Hispanic ethnicity, no. (%)	3(21.4)	1(7.1)	4(14.3)
Race, no. (%)			
White	11(78.6)	9(64.3)	20(71.4)
Black/African‐American	0(0)	1(7.1)	1(3.6)
American Indian/Alaska Native	0(0)	1(7.1)	1(3.6)
Asian	2(14.3)	3(21.4)	5(17.9)
Other/multiracial	1(7.1)	0(0)	1(3.6)
AUD symptoms, DSM‐5			
Past year	0.4(1.1)	2.9(2.5)	1.3(2.1)
Lifetime	1.1(1.6)	4.0(2.1)	2.1(2.3)
AUDIT score	3.0(1.0)	11.8(4.9)	7.4(5.6)
AUDIT‐C score	2.9(1.0)	7.3(1.3)	5.1(2.5)
Alcohol consumption (past 90 days)			
Drinks/drinking day	2.2(1.1)	4.7(2.6)	3.5(2.3)
Drinking days, %	23.2(11.2)	40.8(22.3)	32.0(19.5)
Heavy drinking days	1.8(3.5)	15.5(11.8)	8.6(11.0)
Alcohol craving (PACS)	1.8(3.0)	6.8(5.2)	4.3(4.9)

*Note:* This table summarizes demographic, diagnostic and alcohol‐related variables for participants in the low‐risk (low drinking) and high‐risk (high drinking) alcohol consumption groups, as well as the total sample (*N* = 28). Each group included an equal number of males and females (*n* = 7 per sex). Values are presented as mean (standard deviation [SD]) for continuous variables or number (percentage [%]) for categorical variables.

DSM‐5 AUD symptom counts were assessed using the Structured Clinical Interview for DSM‐5 (SCID‐5) [28]. Alcohol use patterns over the past 90 days were assessed via the Timeline Follow‐Back (TLFB) interview [29]. AUDIT and AUDIT‐C scores were used to index alcohol use severity [3], and alcohol craving was measured using the Penn Alcohol Craving Scale (PACS) [30].

### 2.3 Laboratory Sample Collection

Blood samples were collected at a single laboratory visit following baseline questionnaires. Participants were instructed to abstain from alcohol and recreational drugs 24 h before the session. Participants arrived to the lab between 8 and 10 AM. Abstinence from alcohol was confirmed with a breathalyser (BACtrack S80). Baseline demographic information and questionnaire data (including additional measures not presented here) were collected via REDCap survey. Blood samples were collected in sodium heparin‐coated Vacutainer Plastic Tubes (BD, Cat. No. 367878) and were immediately put on ice and processed within 15 min of collection [31, 32].

### 2.4 Biomarker Profiling and Statistical Analysis Strategy

In this study, we investigated blood‐based immune mediators associated with alcohol use severity by analysing whole blood samples from 28 participants. For each participant, we measured 14 immune‐related mediators using a Luminex multiplex immunoassay on both the supernatant and cell lysate components. The results from these two components were then combined to provide a comprehensive immune profile that reflects both intracellular and extracellular immune signalling [32].

We evaluated immune biomarker differences between high‐risk and low‐risk alcohol use groups and assessed their associations with AUDIT and AUDIT‐C scores using both linear regression and random forest models. To examine the diagnostic potential of key biomarkers, we performed ROC analysis. PCA was used to explore broader immune coordination patterns related to alcohol use severity. Additionally, we applied MMR to test for interactions between IL‐1β and other biomarkers in predicting alcohol use scores. Finally, exploratory linear regression analyses were conducted within each risk group separately to clarify the strength of IL‐1β associations with AUDIT and AUDIT‐C scores among low‐risk versus high‐risk individuals.

### 2.5 Blood Cell Lysate and Supernatant Preparation

Blood cell lysate and supernatant preparation were performed as previously described [31, 32]. See Supporting Information for further details on this method.

### 2.6 Luminex Multiplex Immunoassays

Luminex immunoassays were performed as previously described [32]. This method allows simultaneous quantification of biomarkers in both cell lysates and supernatants, providing a comprehensive whole blood profile. A custom 14‐plex magnetic bead‐based kit (ProcartaPlex, Thermo Fisher Scientific, Catalogue #PPX‐14‐MXCE7DR) was used to measure IL‐1β, IL‐3, IL‐5, IL‐6, IL‐7, IL‐8, IL‐17A, IL‐18, TNF‐α, MCP‐1, MIP‐1β, CCL11, BDNF and HMGB1. Full details are available in the Supporting Information.

## 3 Statistical Analysis

To facilitate comparison of participants with high versus low alcohol risk, participants were categorized into one of two groups based on the presence of (1) reported at‐risk drinking in the past month (see inclusion criteria) and (2) elevated AUDIT score, using a cutoff of 6 or greater to indicate higher‐risk drinking (*N* = 20 with AUDIT ≥ 6 [10 female, 10 male], *N* = 20 with AUDIT < 6 [13 female, 7 male]). This threshold was chosen for practical reasons: Based on the distribution of AUDIT scores in this sample, the ≥ 6 cutoff allowed for balanced group sizes and aligned with the exploratory nature of the study. The traditional AUDIT cutoff score of 8+ [25] was also investigated as a secondary analysis in ROC analyses. To ensure sex‐balanced groups, prior to analysis, the sample size for each stratum was reduced to match the smallest group (seven males with AUDIT < 6). Seven samples were randomly selected from each stratum, resulting in a total sample size of *N* = 28 for analysis.

All statistical analyses were performed in R (v4.4.3) using RStudio (v2024.12.1).

### 3.1 Kruskal–Wallis Test

Group differences in biomarker levels by AUDIT score (≥ 6 vs. < 6) and participant sex (male vs. female) were assessed using Kruskal–Wallis tests, as biomarker distributions did not meet normality assumptions (Shapiro–Wilk *p* < 0.05). To explore potential interaction effects between group and sex, stratified analyses were performed where appropriate, although these analyses were considered exploratory due to limited sample size. *H* statistics and *p* values were computed for each comparison. To account for multiple comparisons, false discovery rate (FDR) correction was applied using the Benjamini–Hochberg method. Statistical significance was defined as FDR‐adjusted *p* ≤ 0.05.

Effect sizes for group differences in biomarker levels (AUDIT ≥ 6 vs. < 6) were estimated using Cohen's *d*, with 95% confidence intervals computed analytically using the cohen.d() function in the effsize R package. Effect sizes were interpreted as small (0.20–0.49), medium (0.50–0.79) or large (≥ 0.80).

### 3.2 Linear Regression

Univariate general linear regression models were used to assess whether individual biomarker levels predicted AUDIT and AUDIT‐C scores. For each model, the coefficient of determination (*R* ^2^), raw *p* values and FDR‐adjusted *p* values were reported. *R* ^2^ represents the proportion of variance in AUDIT or AUDIT‐C scores explained by the biomarker. Higher *R* ^2^ values indicate better model fit. Associations were considered statistically significant at FDR‐adjusted *p* ≤ 0.05.

To examine the influence of extreme values, sensitivity analyses were conducted using outlier removal. Outliers were identified as observations exceeding 6.5 times the median absolute deviation (MAD) from the group median, a robust and conservative threshold that minimizes the influence of extreme deviations while preserving the overall distribution of the data.

To evaluate the validity of the regression assumptions, diagnostic plots were generated for each biomarker model. Residuals versus fitted values were plotted to assess linearity and homoscedasticity, and Q–Q plots were used to evaluate the normality of residuals.

### 3.3 Random Forest Model

Random forest model was applied to explore the relative importance of 14 biomarkers in predicting AUDIT and AUDIT‐C scores, with the goal of identifying those contributing most strongly to variance in these outcomes rather than building formal predictive models.

Each model consisted of 1000 decision trees, with each tree recursively splitting the dataset based on biomarker levels to minimize node impurity. Each tree was trained on a bootstrapped sample of the data and learned recursive decision rules that minimized impurity (e.g., variance) at each node based on biomarker thresholds. At each split, a random subset of predictors was selected using the formula: *m*_try_ = √*p*, where *m*_try_ is the number of predictors at each split and *p* is the total number of predictors. This random feature selection introduces variability across trees and reduces overfitting by ensuring that no single predictor dominates the model structure. Predictions were generated by averaging the output of all 1000 trees, resulting in a stable ensemble estimate of AUDIT or AUDIT‐C scores.

To address the modest sample size (*n* = 28) and mitigate sampling variability, two procedures were implemented. First, the random forest process was repeated 100 times using a fixed seed (123). Although the seed remained constant, each run generated a unique forest via internal bootstrapping and feature subsampling. Variable importance scores, mean decrease accuracy (MDA) and mean decrease Gini (MDG), along with performance metrics (*R* ^2^ and root mean square error [33]), were averaged across runs to yield stable estimates of internal model performance.

Second, fivefold cross‐validation was used to evaluate generalizability. This procedure was also repeated 100 times using the same seed. In each iteration, models were trained on fourfolds and tested on the fifth, such that every observation was used as a test case once per run. *R* ^2^, RMSE, MDA and MDG were averaged across all repetitions to summarize cross‐validated results.

*R* ^2^ reflects the proportion of variance in AUDIT or AUDIT‐C scores explained by the model, with higher values indicating better fit. RMSE represents the average prediction error, with lower values indicating greater accuracy. MDA captures the reduction in predictive accuracy when a variable is permuted, while MDG reflects the total decrease in node impurity attributable to that variable across the forest.

Random forest was used in addition to linear regression to evaluate which biomarkers were most informative for predicting AUDIT and AUDIT‐C scores. While linear models assume linear, additive effects and rely on distributional assumptions (e.g., normality and homoscedasticity), random forest is nonparametric and can model nonlinear patterns and higher order interactions without requiring explicit specification. This flexibility allows random forest to capture predictive signals that may be overlooked in linear regression. By using both methods, we combined the interpretability of linear models with the pattern‐detection capabilities of ensemble learning, enabling a more comprehensive assessment of biomarker contributions.

This framework aligns with prior applications of random forest in small biological datasets [34] and supports its use for exploratory biomarker evaluation in modestly sized human studies.

### 3.4 ROC Analysis

ROC curve analysis was used to evaluate the discriminatory ability of IL‐1β to classify participants based on several thresholds. These included AUDIT scores greater than or equal to 6 versus less than 6, AUDIT scores greater than or equal to 8 versus less than 8 and AUDIT‐C scores—defined as scores of 3 or more for females and 4 or more for males versus below these thresholds. For each comparison, the area under the curve (AUC), optimal threshold based on Youden's index, sensitivity (true positive rate [TPR]), specificity and false positive rate [FPR] (1—specificity) were calculated. AUC values were interpreted using standard discrimination categories, where 0.5 indicates no discrimination, 0.6–0.7 indicates poor discrimination, 0.7– 0.8 indicates fair discrimination, 0.8–0.9 indicates good discrimination and values of 0.9 or above indicate excellent discrimination. All ROC curves and thresholds were generated using the pROC package in R.

### 3.5 MMR

MMR was used to assess whether the interaction between IL‐1β and each of the other 13 biomarkers predicted AUDIT or AUDIT‐C scores. Each model included IL‐1β, the target biomarker and their interaction term. All predictors were mean‐centred to reduce multicollinearity. Models were estimated using ordinary least squares (OLS), and FDR correction was applied to interaction term *p* values. Interactions were considered significant at adj *p* ≤ 0.05.

### 3.6 PCA

PCA is a dimensionality reduction technique that captures the most significant sources of variance within a dataset [35]. We used PCA to explore overall patterns of biomarker covariation and to identify which biomarkers contributed most to the primary axes of variance. This approach helps visualize group‐level differences and potential biomarker clusters in a multivariate space. Given the relatively small sample size (*n* = 28), this analysis is exploratory. PCA was conducted separately in high‐risk (AUDIT ≥ 6) and low‐risk (AUDIT < 6) groups to characterize patterns of biomarker variability and identify principal contributors to PC1 and PC2. Analyses were performed on *z*‐score normalized values for all 14 biomarkers. The top two contributors to PC1 and PC2 were identified based on squared loadings. PCA biplots were generated with cos^2^‐based colour gradients to visualize the magnitude and orientation of each variable's contribution.

Reproducible R code, including packages and functions used, is provided in the Supporting Information.

## 4 Results

Data from 28 adults were analysed (Table 1), with equal numbers in the low‐risk (*n* = 14) and high‐risk (*n* = 14) groups. Each group included seven males and seven females. Participants were predominantly White, with additional representation from Asian, Black/African American, American Indian/Alaska Native, Hispanic and multiracial backgrounds. The average age was 26.5 years.

Clear group differences emerged in alcohol use patterns and related clinical measures. Compared with the low‐risk group, participants in the high‐risk group reported more drinks per occasion, drank on a greater proportion of days and had more heavy drinking days. Self‐reported craving, assessed using the PACS, was also higher in the high‐risk group (Table 1). These results confirm distinct behavioural and clinical profiles, supporting the group‐based comparison of immune biomarkers in subsequent analyses.

### 4.1 IL‐1β, IL‐18, IL‐7 and CCL11 Levels Are Elevated in Whole Blood From Participants in the High‐Risk Group

Using Luminex multiplex immunoassays, we measured levels of BDNF, CCL11, HMGB1, IL‐1β, IL‐3, IL‐5, IL‐6, IL‐7, IL‐8, IL‐17A, IL‐18, MCP‐1, MIP‐1β and TNF‐α in whole blood samples (combined cell lysates and supernatants) from the low‐risk (*n* = 14) and high‐risk (*n* = 14) groups.

Group and sex differences were assessed using the Kruskal–Wallis test, with stratified analyses conducted to examine potential Group × Sex interactions. FDR correction was applied to account for multiple comparisons.

**FIGURE 1:**
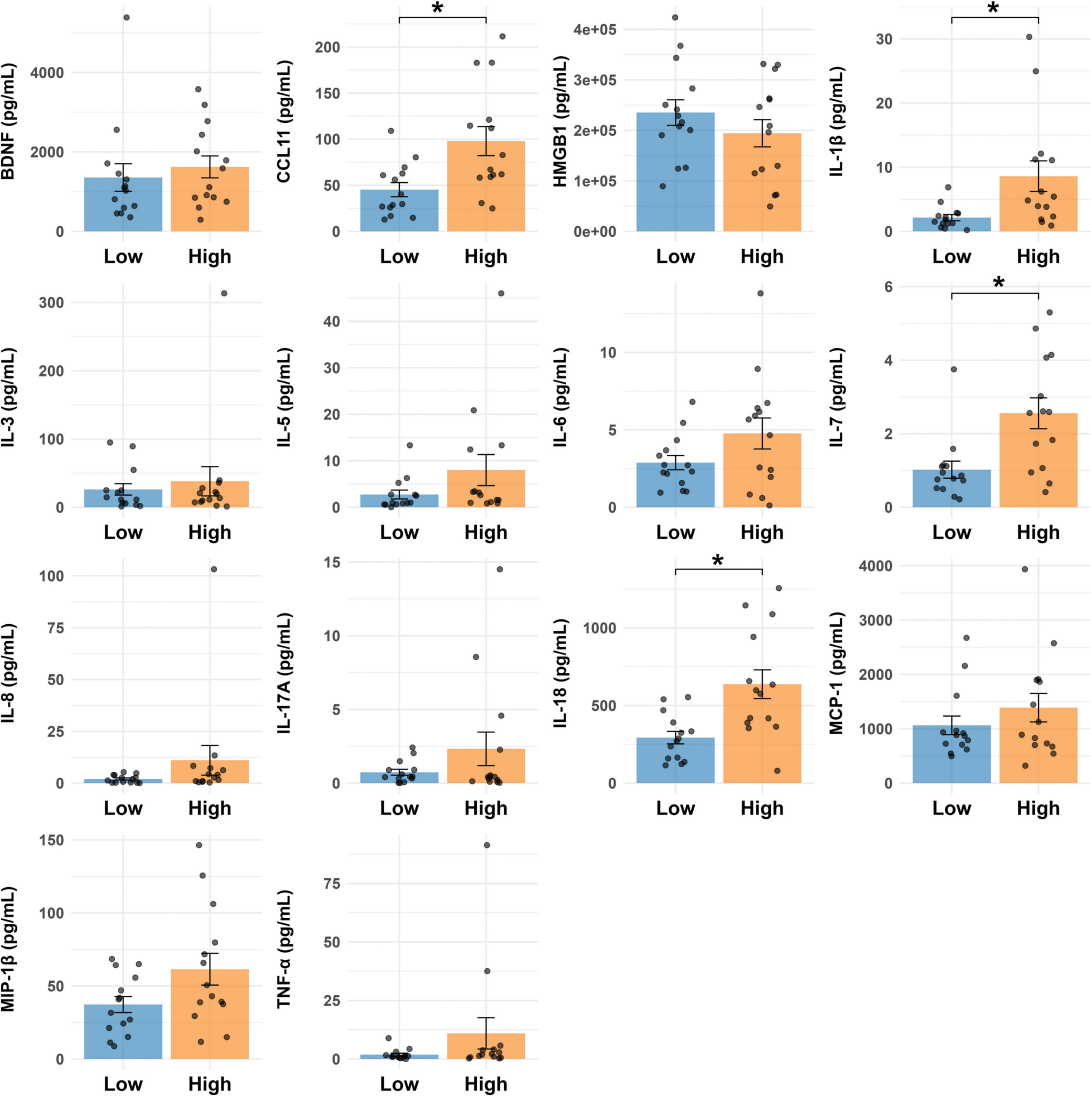
Elevated levels of IL‐1β, IL‐18, IL‐7 and CCL11 in individuals with AUDIT scores ≥ 6 compared with AUDIT scores < 6. Biomarker levels (pg/mL) were measured in whole blood samples (cell lysates + supernatants) from 28 participants using Luminex multiplex immunoassays. Participants were grouped by AUDIT score, with 14 individuals in the AUDIT ≥ 6 group and 14 in the AUDIT < 6 group. Each group included both males (*n* = 7/group) and females (n = 7/group). Significantly higher levels of IL‐1β, IL‐18, IL‐7 and CCL11 were observed in the AUDIT ≥ 6 group compared with the AUDIT < 6 group. No significant differences were found for BDNF, HMGB1, IL‐3, IL‐5, IL‐6, IL‐8, IL‐17A, MCP‐1, MIP‐1β or TNF‐α. Group comparisons were made using the Kruskal–Wallis test, with false discovery rate (FDR) correction applied for multiple comparisons. Bars represent group means with standard error, and individual data points (*n* = 14 per group) are plotted directly on the bars. Asterisks indicate statistical significance at FDR‐adjusted *p* < 0.05.

Significant elevations in CCL11 (116.2 ± 50.4%, *H* = 7.1, df = 1, *p* = 0.008, adj *p* = 0.03), IL‐1β (301.9 ± 143.1%, *H* = 7.9, df = 1, *p* = 0.005, adj *p* = 0.03), IL‐7 (150.3 ± 70.1%, *H* = 7.6, df = 1, *p* = 0.006, adj *p* = 0.03) and IL‐18 (117.3 ± 43.5%, *H* = 9.2, df = 1, *p* = 0.002, adj *p* = 0.03) were observed in the high‐risk group compared with the low‐risk group (Figure 1).

In contrast, no significant group differences were observed for BDNF, HMGB1, IL‐3, IL‐5, IL‐6, IL‐8, IL‐17A, MCP‐1, MIP‐1β or TNF‐α following FDR correction (adj *p* > 0.05; Figure 1; Table S1). No significant main effects of sex or Group × Sex interactions were found for any biomarker (adj *p* > 0.05; Table S1). However, these exploratory analyses of Group × Sex interactions were underpowered due to the modest sample size and should be interpreted cautiously.

To quantify the magnitude of group differences, Cohen's *d* was calculated. Large effect sizes were observed for IL‐18 (*d* = 1.25, 95% CI: 0.42–2.07), IL‐7 (*d* = 1.17, 95% CI: 0.36–1.99), IL‐1β (*d* = 0.97, 95% CI: 0.18–1.77) and CCL11 (*d* = 1.10, 95% CI: 0.29–1.91) (Table S1).

### 4.2 IL‐1β Is Identified as the Strongest Predictor of Both AUDIT and AUDIT‐C Scores

Linear regression and random forest analyses were conducted on the full dataset (*n* = 28) to evaluate associations between 14 immune‐related biomarkers and alcohol use severity. Linear regression was used to evaluate individual associations with AUDIT and AUDIT‐C scores, while random forest provided a complementary nonlinear method to assess the relative importance of each biomarker. For linear regression, model assumptions were validated via residual diagnostics, and statistical significance was determined using FDR‐adjusted *p* values (≤ 0.05). Sensitivity analyses were performed after outlier removal using a conservative 6.5× MAD threshold.

**FIGURE 2:**
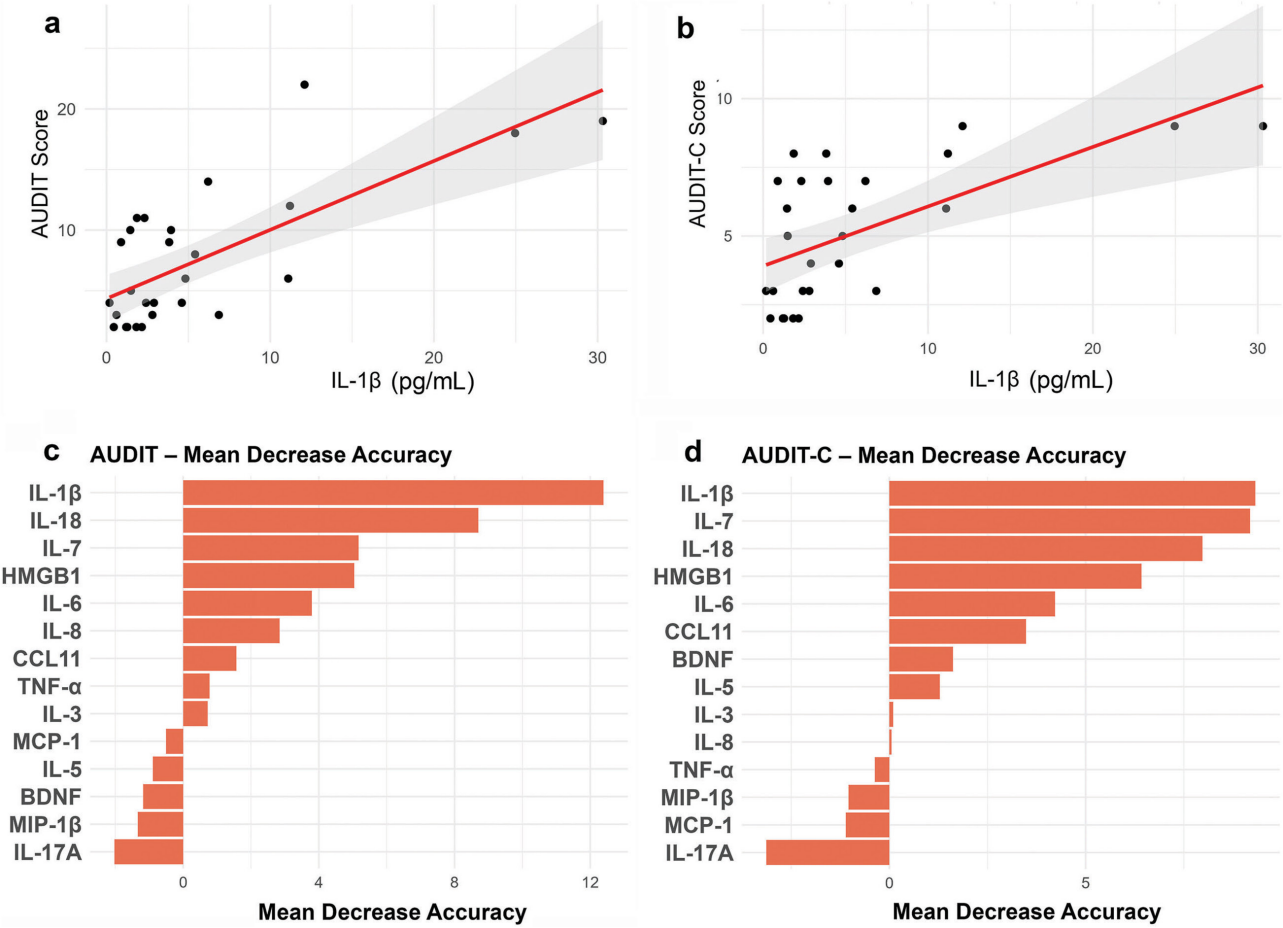
IL‐1β is the strongest predictor of AUDIT and AUDIT‐C scores in both linear regression and random forest models. (a,b) Linear regression analyses showing significant positive associations between IL‐1β levels and both AUDIT (a) and AUDIT‐C (b) scores. Each point represents an individual participant (*n* = 28). Red lines indicate fitted regression lines; shaded grey areas represent 95% confidence intervals. IL‐1β explained the largest proportion of variance in both AUDIT (*R* ^2^ = 0.51, adj *p* = 0.0002) and AUDIT‐C (*R* ^2^ = 0.38, adj *p* = 0.007) scores. These associations remained statistically significant after FDR correction and outlier removal. (c,d) Variable importance plots from random forest models predicting AUDIT (c) and AUDIT‐C (d) scores based on biomarker levels. Bars represent mean decrease in accuracy (MDA), where higher values indicate greater predictive importance. IL‐1β ranked as the most influential predictor for both outcomes. For AUDIT, it was followed by IL‐18, IL‐7, HMGB1, IL‐6 and IL‐8. For AUDIT‐C, IL‐1β and IL‐7 had nearly equal importance, followed by IL‐18, HMGB1, IL‐6 and CCL11. In both models, biomarkers such as IL‐17A, MIP‐1β and MCP‐1 contributed minimally or negatively to prediction accuracy.

For AUDIT scores, linear regression identified IL‐1β as the strongest individual biomarker, explaining 51% of the variance (*R* ^2^ = 0.51, adj *p* = 0.0002; Figure 2a). After outlier removal, IL‐1β remained significant (*R* ^2^ = 0.34, adj *p* = 0.02), indicating that the effect was not driven solely by extreme values (Table S2). No other biomarkers reached significance after FDR correction. Model assumptions were met, with residual plots indicating linearity, homoscedasticity and near‐normal distribution (Figure S1). For AUDIT‐C scores, IL‐1β again showed the strongest association (*R* ^2^ = 0.38, adj *p* = 0.007; Figure 2b), followed by IL‐7 (*R* ^2^ = 0.33, adj *p* = 0.009) and CCL11 (*R* ^2^ = 0.25, adj *p* = 0.03). After outlier removal, IL‐1β (*R* ^2^ = 0.30, adj *p* = 0.03), IL‐7 (*R* ^2^ = 0.30, adj *p* = 0.03) and CCL11 (*R* ^2^ = 0.25, adj *p* = 0.03) remained significantly associated with AUDIT‐C scores (Table S2). Residual diagnostics for these models confirmed no major deviations from linearity or normality (Figure S2).

The random forest model for AUDIT scores demonstrated high internal fit (*R* ^2^ = 0.93 ± 0.003; RMSE = 2.07 ± 0.03), with IL‐1β showing the highest variable importance (MDA = 12.4; MDG = 178.1), followed by IL‐18 (MDA = 8.7; MDG = 95.0), IL‐7 (MDA = 5.2; MDG = 54.1), HMGB1 (MDA = 5.0; MDG = 53.5), IL‐6 (MDA = 3.8; MDG = 67.4) and IL‐8 (MDA = 2.8; MDG = 86.0). CCL11 also contributed (MDA = 1.6; MDG = 52.0), while TNF‐α, IL‐3, MCP‐1, IL‐5, BDNF, MIP‐1β and IL‐17A had minimal or negative importance (Figure 2c). In fivefold cross‐validation repeated 100 times, the model achieved a mean *R* ^2^ of 0.50 and RMSE of 4.5, with standard deviation (SD) = 0 across all repetitions. IL‐1β remained the top predictor (MDA = 9.9; MDG = 125.5), followed by IL‐18 (MDA = 7.2; MDG = 69.6), IL‐7 (MDA = 3.5; MDG = 42.1), IL‐6 (MDA = 3.5; MDG = 52.5), HMGB1 (MDA = 2.2; MDG = 44.6), CCL11 (MDA = 2.1; MDG = 39.6) and IL‐8 (MDA = 1.4; MDG = 66.3), while other biomarkers continued to show low or negative importance (Figure S3a).

For AUDIT‐C scores, the random forest model demonstrated similarly high internal fit (*R* ^2^ = 0.94 ± 0.003; RMSE = 0.91 ± 0.01), with IL‐1β again ranking highest in importance (MDA = 9.3; MDG = 22.6), followed closely by IL‐7 (MDA = 9.2; MDG = 20.9), IL‐18 (MDA = 8.0; MDG = 19.0) and HMGB1 (MDA = 6.4; MDG = 11.7). IL‐6 (MDA = 4.2; MDG = 14.6) and CCL11 (MDA = 3.5; MDG = 14.4) also contributed meaningfully, while BDNF, IL‐5, IL‐3, IL‐8, TNF‐α, MIP‐1β, MCP‐1 and IL‐17A showed limited or negative contributions (Figure 2d). In fivefold cross‐validation, the mean *R* ^2^ was 0.46 and RMSE was 2.1, with SD = 0. IL‐1β and IL‐7 showed nearly identical Mean Decrease Accuracy values (IL‐1β: MDA = 8.0; IL‐7: MDA = 8.2) and similar Mean Decrease Gini scores (IL‐1β: MDG = 16.9; IL‐7: MDG = 14.5), followed by IL‐18 (MDA = 6.4; MDG = 14.6), HMGB1 (MDA = 4.0; MDG = 9.2), CCL11 (MDA = 3.3; MDG = 11.2) and IL‐6 (MDA = 2.5; MDG = 11.0). IL‐5 (MDA = 1.4; MDG = 5.6) and BDNF (MDA = 1.3; MDG = 6.7) also contributed modestly, while IL‐8, TNF‐α, IL‐3, MCP‐1, MIP‐1β and IL‐17A continued to show minimal or negative importance (Figure S3b).

### 4.3 Predictive Value of IL‐1β for Elevated AUDIT and AUDIT‐C Scores

**FIGURE 3:**
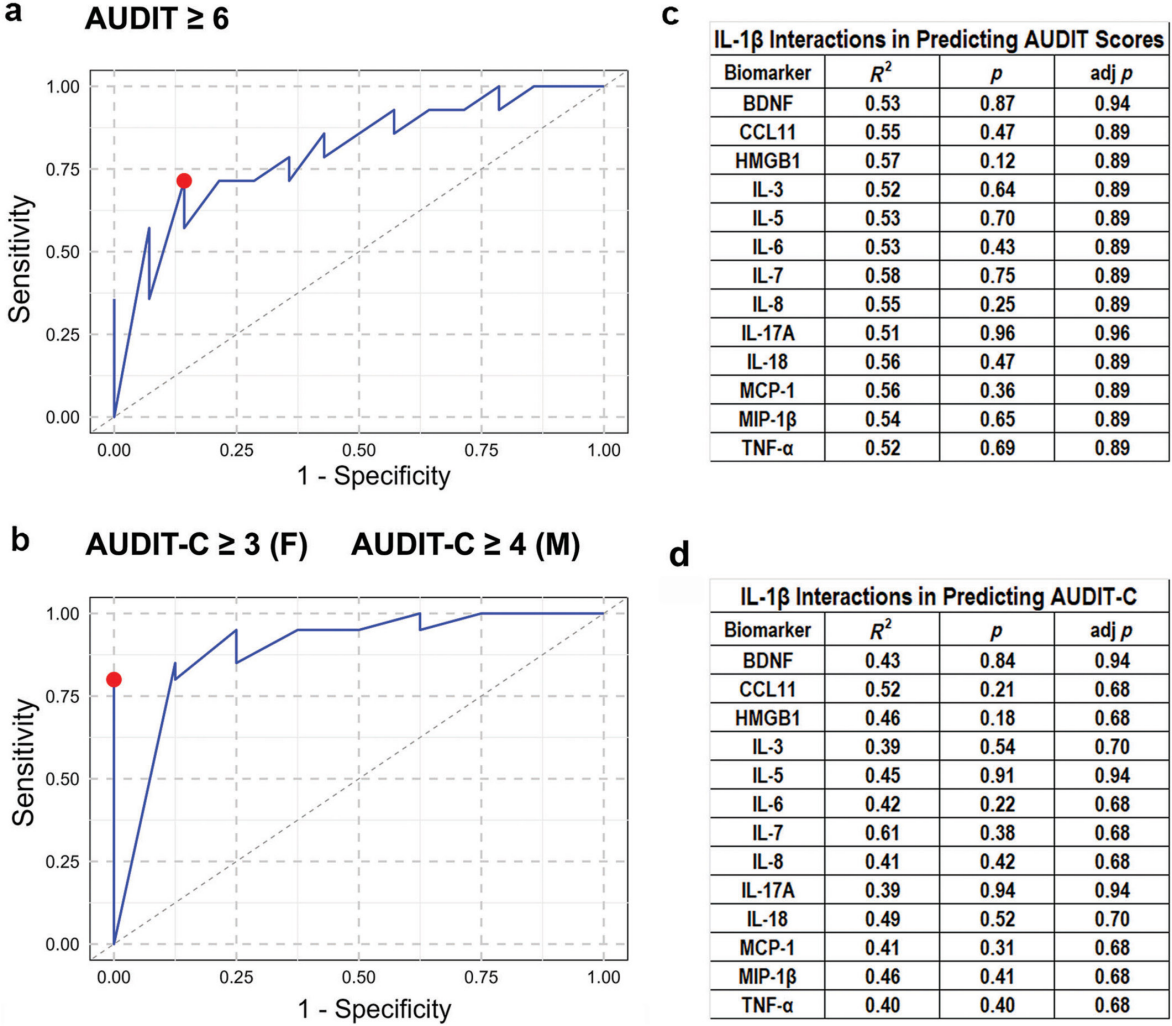
Diagnostic potential of IL‐1β based on AUDIT and AUDIT‐C thresholds, and its independent predictive value. (a,b) Receiver operating characteristic (ROC) curve analyses were conducted to evaluate the ability of IL‐1β to classify individuals based on AUDIT and AUDIT‐C thresholds. In (a), classification was based on an AUDIT score of ≥ 6; in (b), on AUDIT‐C thresholds of ≥ 3 for females (F) and ≥ 4 for males (M). IL‐1β showed strong classification performance in both models, with optimal thresholds (red dots) identified: 3.36 pg/mL for AUDIT and 2.24 pg/mL for AUDIT‐C. (c,d) Moderated multiple regression (MMR) analyses were conducted to evaluate whether IL‐1β interacts with any of the 13 other measured biomarkers in predicting continuous AUDIT (c) and AUDIT‐C (d) scores. No significant interaction effects were observed after FDR correction (adj *p* > 0.05), indicating that IL‐1β predicts both outcomes independently of other biomarkers.

IL‐1β demonstrated potential as a biomarker for elevated AUDIT and AUDIT‐C scores, effectively distinguishing participants with higher versus lower alcohol use severity. For an AUDIT score ≥ 6, the AUC was 0.81, indicating good discrimination, with an optimal threshold of 3.36 pg/mL, yielding a sensitivity (TPR) of 0.71, specificity of 0.86 and FPR of 0.14 (Figure 3a). At a higher AUDIT cutoff (≥ 8), the AUC decreased to 0.74, indicating fair discrimination, with the same threshold (3.36 pg/mL), a sensitivity (TPR) of 0.67, specificity of 0.75 and FPR of 0.25.

Regarding the AUDIT‐C, IL‐1β exhibited excellent discrimination, achieving an AUC of 0.94. The optimal threshold was 2.24 pg/mL, corresponding to a sensitivity (TPR) of 0.80, a specificity of 1.00 and FPR of zero (Figure 3b).

### 4.4 Predictive Validity of IL‐1β Was Independent of Other Biomarkers

To evaluate whether IL‐1β independently predicts AUDIT and AUDIT‐C scores apart from the other 13 studied biomarkers, MMR was used to assess interaction effects between IL‐1β and the 13 biomarkers in predicting AUDIT and AUDIT‐C scores. FDR correction was applied to adjust for multiple comparisons. The results demonstrated that IL‐1β independently predicts both AUDIT (Figure 3c) and AUDIT‐C (Figure 3d) scores, with none of the tested biomarkers showing significant interaction effects with IL‐1β. All *p* values and FDR adjusted *p* values were above 0.05, indicating that IL‐1β's influence on AUDIT and AUDIT‐C scores is independent rather than interaction driven.

### 4.5 PCA Reveals Distinct Biomarker Profiles Between High‐Risk and Low‐Risk Groups

PCA was performed to examine biomarker variability and identify features distinguishing participants in the high‐risk (AUDIT ≥ 6) and low‐risk (AUDIT < 6) groups. PCA biplots showed clear group separation based on vector orientation, length and distribution.

**FIGURE 4:**
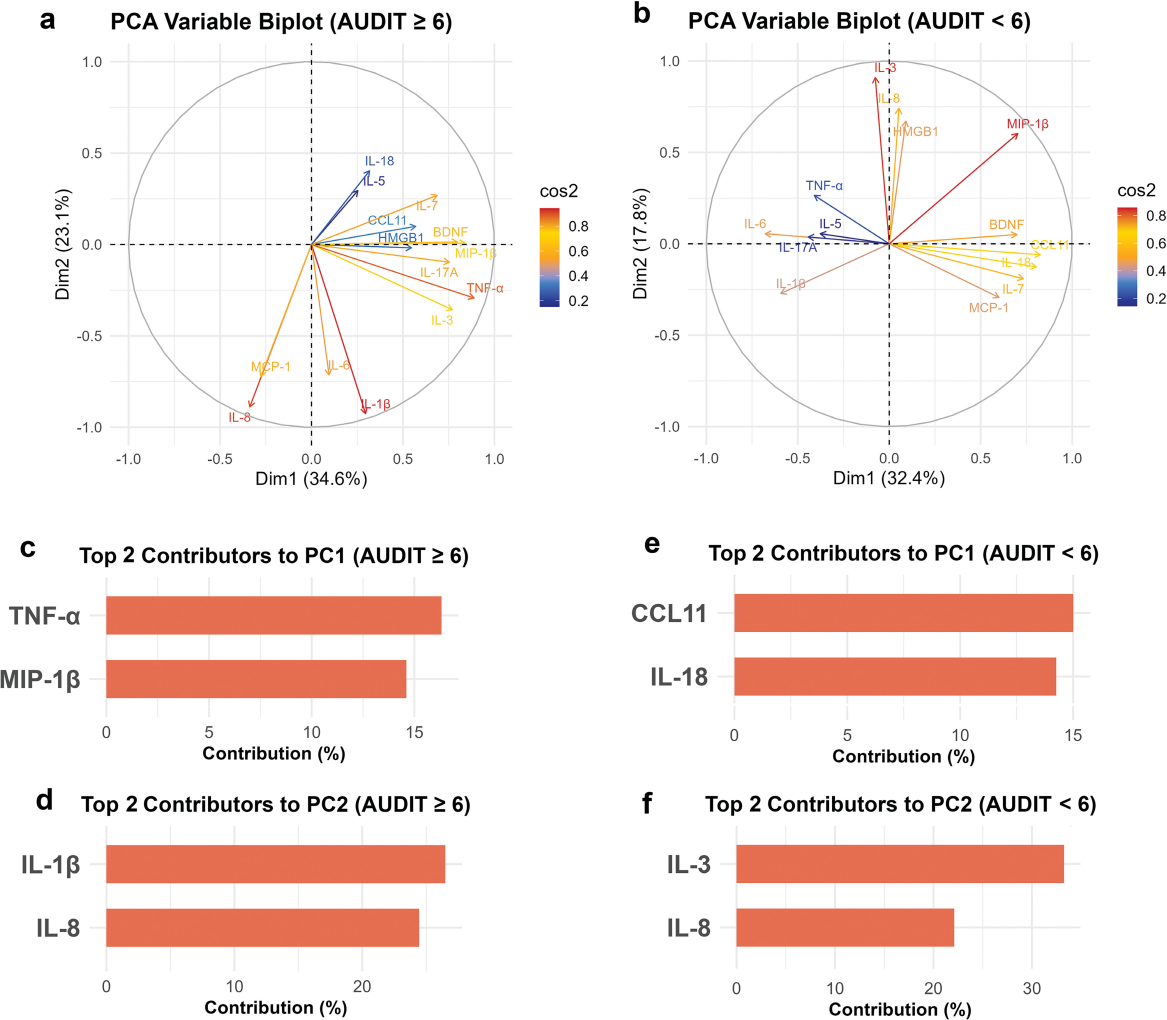
Principal component analysis (PCA) reveals distinct biomarker profiles between high‐risk and low‐risk AUDIT score groups. (a,b) PCA variable biplots showing the distribution and contribution of biomarkers to the first two principal components, PC1 (Dim1) and PC2 (Dim2), in participants with AUDIT ≥ 6 (a) and AUDIT < 6 (b). Dim1 and Dim2 refer to the first and second principal components, which together capture the greatest variance in the dataset. For the AUDIT ≥ 6 group (high risk), Dim1 and Dim2 account for 34.6% and 23.1% of the total variance, respectively. In the AUDIT < 6 group (low risk), Dim1 and Dim2 explain 32.4% and 17.8% of the variance. In the high‐risk group, vectors for IL‐1β, IL‐8, MCP‐1, TNF‐α and MIP‐1β are longer and more dispersed, indicating higher interindividual variability and a more heterogeneous inflammatory profile. In contrast, the low‐risk group displays more compact and aligned vectors, reflecting a more coordinated and stable immune response. Vector colour corresponds to the cos^2^ value, shown as a blue‐to‐red gradient, with warmer colours indicating greater contribution to the principal components. (c,d) Top two contributors to PC1 and PC2 in the AUDIT ≥ 6 group. PC1 was primarily driven by TNF‐α and MIP‐1β, while PC2 was dominated by IL‐1β and IL‐8. (e,f) Top two contributors to PC1 and PC2 in the AUDIT < 6 group. PC1 was shaped mainly by CCL11 and IL‐18, whereas PC2 was driven by IL‐3 and IL‐8.

In the high‐risk group, biomarkers such as IL‐1β, IL‐8, MCP‐1, TNF‐α and MIP‐1β showed greater dispersion and vector length, reflecting high interindividual variability and strong contributions to the first two principal components, suggesting an uncoordinated inflammatory response (Figure 4a). Vector colour gradients further highlighted IL‐1β, IL‐8, TNF‐α, MCP‐1, IL‐6, IL‐7 and MIP‐1β as dominant contributors, while IL‐5 and HMGB1 had minimal influence.

In contrast, the low‐risk group displayed a more compact and coordinated biomarker pattern, with vectors such as CCL11, IL‐18, IL‐7, BDNF and MCP‐1 pointing in similar directions and showing shorter lengths, indicating lower variability and a more regulated immune profile (Figure 4b). Most vectors appeared yellow to orange, reflecting moderate contributions; IL‐5, TNF‐α and IL‐17A showed minimal influence (blue), while MIP‐1β and IL‐3 stood out (red) for their variability.

PC loadings further supported these findings: In the high‐risk group, PC1 was driven by TNF‐α and MIP‐1β (Figure 4c) and PC2 by IL‐1β and IL‐8 (Figure 4d). In the low‐risk group, PC1 was shaped by CCL11 and IL‐18 (Figure 4e) and PC2 by IL‐3 and IL‐8 (Figure 4f). These results underscore distinct patterns of biomarker coordination between groups.

## 5 Discussion

In this study, we found that IL‐1β emerged as a promising and independent candidate biomarker of elevated AUDIT scores, among 14 immune‐related mediators measured in whole blood, including cytokines, chemokines, HMGB1 and BDNF. Our results showed that IL‐1β, IL‐18, IL‐7 and CCL11 were significantly elevated in the high‐risk group, with large effect sizes (Cohen's *d*) that remained significant after FDR correction. These findings suggest a proinflammatory immune profile associated with greater levels of consumption and elevated AUDIT scores. Unlike studies measuring only circulating inflammatory mediators, we assessed both intracellular and extracellular mediator levels by analysing cell lysates and supernatants from whole blood, providing a more comprehensive view of immune activation. The observed elevations may reflect increased production of proinflammatory mediators or altered immune cell function. While we did not assess leukocyte subtypes, our findings align with prior evidence of heightened immune activity in individuals at elevated risk for AUD, such as increased white blood cell counts reported in those with a family history of the disorder [36].

It is important to note that participants in the higher AUDIT group were, on average, in the moderate‐risk range for alcohol use severity, based on both DSM‐5 symptom counts and AUDIT scores (see Table 1) [3]. While these individuals did not meet criteria for severe AUD or very high‐risk drinking, we identified biomarkers that may signal immune changes associated with early or moderate levels of risk, where interventions may be most effective [17]. The observed elevations in IL‐1β and its strong association with AUDIT and AUDIT‐C scores suggest that immune dysregulation can be detected even in individuals who do not meet full diagnostic criteria for AUD [18]. These findings support the potential utility of IL‐1β as a biomarker to identify individuals with elevated risk, a group with increased likelihood for progression to more severe alcohol‐related problems.

To evaluate associations between immune‐related mediators and alcohol use severity, we used both linear regression and random forest models, with random forest serving as a complementary, nonlinear method. Linear regression analysis demonstrated that IL‐1β significantly predicted both AUDIT and AUDIT‐C scores. In contrast, although IL‐7, IL‐18 and CCL11 were elevated in the high‐risk group, only IL‐7 and CCL11 showed significant associations with AUDIT‐C scores, while none were significantly associated with AUDIT scores. This distinction supports the idea that IL‐1β may be related to a broader spectrum of alcohol‐related outcomes, while IL‐7 and CCL11 may reflect consumption‐specific immune changes. Given that AUDIT‐C focuses solely on alcohol intake, while AUDIT includes hazardous use and dependence symptoms [3, 4], the consistent signal from IL‐1β underscores its relevance to both behavioural and physiological dimensions of alcohol use.

Random forest analyses confirmed IL‐1β as the most important predictor for both AUDIT and AUDIT‐C scores, ranking highest in variable importance across full and cross‐validation models. For AUDIT, IL‐18, IL‐7, HMGB1, IL‐6, IL‐8 and CCL11 also demonstrated important contributions, while mediators such as TNF‐α, IL‐3, MCP‐1, IL‐5, BDNF, MIP‐1β and IL‐17A showed minimal or negative importance. However, in linear regression, only IL‐1β was significantly associated with AUDIT scores, while the other random forest important predictors did not reach statistical significance. For AUDIT‐C, in addition to IL‐1β, IL‐7, IL‐18, HMGB1, IL‐6 and CCL11 were important contributors in the random forest analysis. In contrast, linear regression identified IL‐1β, IL‐7 and CCL11 as significantly associated with AUDIT‐C scores, while IL‐18, HMGB1 and IL‐6 did not meet the FDR‐adjusted threshold. Thus, although a high number of the examined immune mediators did not reach significance in linear regression for AUDIT and AUDIT‐C, their consistent ranking in the random forest analysis highlights potential nonlinear or interaction effects that may be overlooked by linear models alone. The convergence between random forest and linear regression strengthens confidence in IL‐1β as a key biomarker, while also demonstrating that machine learning adds value by identifying hidden structure in the data that linear models may overlook.

To further examine the relationship between IL‐1β and alcohol use severity, we conducted separate linear regression analyses within the high‐ and low‐risk groups. In the high‐risk group, IL‐1β was significantly associated with both AUDIT and AUDIT‐C scores, while no significant associations were observed in the low‐risk group (Figure S4). These results suggest that IL‐1β may be particularly relevant in individuals with elevated alcohol‐related risk, supporting its potential utility as a biomarker of immune dysregulation in AUD. MMR analyses, which assess whether the effect of a predictor varies depending on the levels of other variables, further confirmed that IL‐1β functions independently of other measured immune mediators. No significant interaction effects were detected, suggesting that IL‐1β captures a distinct and specific immune signature related to alcohol‐related outcomes. While IL‐1β appears to act independently, future multivariate models incorporating IL‐18, IL‐7, HMGB1, IL‐6, IL‐8, CCL11 and other immune markers alongside IL‐1β may reveal complementary or synergistic immune patterns [37], improving risk stratification and providing a more comprehensive understanding of alcohol‐associated immune alterations.

IL‐1β is produced as an inactive precursor that requires inflammasome‐dependent cleavage by caspase‐1 for activation [38, 39]. Our detergent lysis and sonication protocol likely captured both free and vesicle‐associated forms of IL‐1β, supporting the strong and consistent detection observed. Previous studies have shown that intracellular processing of pro‐IL‐1β and its extracellular release can occur as distinct and uncoupled events [38, 39]. By measuring IL‐1β in both lysate and supernatant from whole blood, our approach provides an integrated and physiologically relevant measure of inflammasome‐dependent IL‐1β processing and release in vivo.

The potential of IL‐1β as a biomarker for elevated AUDIT and AUDIT‐C scores was supported by ROC curve analyses, which demonstrated its ability to effectively distinguish participants with higher versus lower alcohol use severity. Notably, IL‐1β showed excellent specificity and discrimination in AUDIT‐C scores, while performance for the full AUDIT score remained good but slightly lower. This pattern suggests that IL‐1β may more accurately reflect recent alcohol consumption patterns, particularly those captured by AUDIT‐C, which focuses on hazardous use [4]. Given that AUDIT‐C contributed substantially to the total AUDIT score in our cohort and that the sample did not display clear evidence of alcohol‐related harm, the strong association between IL‐1β and AUDIT‐C supports its potential as an early‐stage immune marker of hazardous drinking [17]. This interpretation is further supported by participants' low craving scores and moderate levels of heavy drinking, which suggest patterns of binge or risky alcohol use rather than dependence [27]. It may also serve as a dynamic marker of change in immune responses [40, 41]. In addition, ROC curve performance for IL‐1β was stronger when using the more conservative AUDIT ≥ 6 threshold compared with the traditional ≥ 8 cutoff, supporting the utility of lower thresholds for detecting early immune changes associated with AUD.

IL‐1β is a well‐established proinflammatory cytokine that plays a central role in innate immune responses. It is produced primarily by activated monocytes and macrophages following inflammasome activation and acts as a key mediator of fever, leukocyte recruitment and cytokine cascades [39, 42, 43]. In the context of AUD, IL‐1β has been shown to contribute to both peripheral inflammation and neuroinflammation. Chronic alcohol exposure is known to activate immune cells and inflammasomes, leading to increased production of IL‐1β, which in turn may exacerbate tissue damage and impact mood, cognition and reward‐related neurocircuitry [40, 41, 44]. These known mechanisms align with our findings and support IL‐1β as a candidate biomarker of AUD, based on AUDIT scores.

A comprehensive understanding of alcohol‐related immune dysregulation requires assessing both proinflammatory and anti‐inflammatory mediators. While our study focused on proinflammatory cytokines and chemokines, future work should incorporate regulatory molecules such as interleukin‐1 receptor antagonist (IL‐1RA), which blocks IL‐1β activity at its receptor. The IL‐1β/IL‐1RA ratio has been proposed as a useful index of inflammatory imbalance in the context of substance use, including AUD [45, 46].

In this study, we also measured BDNF, which has been shown to promote IL‐10 production in other systems [47, 48]. However, in our dataset, BDNF levels did not differ between high‐risk and low‐risk groups and were not associated with AUDIT or AUDIT‐C scores. Including anti‐inflammatory markers such as IL‐1RA and IL‐10 in future studies may improve interpretation of elevated proinflammatory signals and provide a more balanced view of immune regulation in alcohol‐exposed individuals.

PCA revealed greater immune heterogeneity among individuals in the high‐risk group, particularly involving proinflammatory cytokines such as IL‐1β and TNF‐α. PCA is a dimensionality reduction technique that identifies the principal components that capture the greatest variance across multiple variables, allowing for visualization of complex multivariate relationships [35]. The immune heterogeneity suggests that chronic alcohol consumption may disrupt immune homeostasis, leading to dysregulated inflammatory responses [1, 14, 49, 50, 51]. In contrast, individuals in the low‐risk group exhibited more stable and coordinated immune profiles, indicating preserved immune regulation. Given the relatively small sample size (*n* = 28), however, the PCA is exploratory in nature, and the identified patterns may not be reproducible in larger cohorts, underscoring the pilot nature of the study.

Notably, TNF‐α was a top contributor to PC1 within the high‐risk group, primarily due to its high inter‐individual variability (Table S3). Conversely, IL‐1β, despite its consistent elevation and robust associations with both AUDIT and AUDIT‐C scores, did not exhibit a similarly high loading on PC1, likely due to its lower variability across participants. This contrast underscores the complexity of immune alterations in alcohol use, where some cytokines like IL‐1β may serve as stable indicators of alcohol‐related immune dysregulation, while others like TNF‐α may reflect more individualized immune responses.

A key methodological strength of this study is the use of whole blood preparations, combining cell lysate and supernatant. This approach allows simultaneous quantification of multiple immune mediators, including soluble, intracellular and vesicle‐encapsulated cytokines, chemokines and damage‐associated molecular patterns, providing a robust and reproducible immune profile from a single sample [32]. Through detergent‐based lysis and sonication, our protocol disrupts extracellular vesicles (EVs) released by monocytes and lymphocytes, capturing biologically active immune mediators that are often underrepresented or undetectable in plasma or serum assays [31, 32]. IL‐1β is known to be secreted via non‐classical pathways, including packaging into EVs during inflammasome activation, where it remains biologically active and promotes inflammation [52, 53, 54]. Our method likely captured both soluble and vesicle‐associated IL‐1β, which may explain its strong and consistent performance across models.

In addition to traditional linear regression, we employed machine learning to objectively rank biomarker importance, highlighting IL‐1β as the top contributor across models. This data‐driven approach illustrates the value of combining complementary analytical strategies to advance biomarker discovery in AUD research. While based on a modest sample size, these findings may hold potential clinical relevance. Although not yet ready for routine clinical application, the combined use of statistical and machine learning models lays the groundwork for translational biomarker development. With further refinement and automation, such approaches hold promise for integration into specialized clinical laboratories to screen, monitor or stratify individuals with AUD or other inflammation‐related conditions [31].

A key limitation of this study is its cross‐sectional design, which precludes distinguishing the inflammatory effects of past alcohol exposure from those related to current use patterns. Although we used the AUDIT and AUDIT‐C approaches, validated screening tools [3, 4, 25, 26, 27], to stratify alcohol‐related risk, future studies should incorporate comprehensive diagnostic assessments in larger samples. We shifted our recruitment strategy to focus on AUDIT and AUDIT‐C scores after observing stronger biomarker signals relative to formal AUD diagnoses early in the study (data not shown). Nonetheless, future research is needed to more directly associate immune biomarkers with DSM‐5‐defined AUD and its severity subtypes.

While we attempted to minimize confounding variables such as smoking and other known contributors to inflammatory signalling, it remains difficult to fully isolate the effects of alcohol use from other environmental and lifestyle factors that may covary with drinking behaviour, including dietary patterns, stress or other substance exposures.

## 6 Conclusion

Our findings demonstrate that IL‐1β is a promising predictor of higher AUDIT scores, a validated clinical indicator of AUD risk. Individuals with elevated AUDIT scores exhibited a distinct immune profile marked by increased proinflammatory mediators and greater immune heterogeneity. These results suggest that immune‐associated markers, particularly IL‐1β, may reflect alcohol‐related immune alterations and serve as potential indicators of heightened AUD risk. Further research in larger, diagnostically defined cohorts is needed to determine the translational potential of these findings for clinical application.

## Author Contributions

Conception and experimental design: A. Leslie Morrow and Christian S. Hendershot. Supervision and funding: A. Leslie Morrow and Christian S. Hendershot. Subject screening, recruitment, instrument administration and blood sample collection: Thomas Gilmore, Michael Bremmer and Todd K. O'Buckley. Laboratory sample analysis: Irina Balan and Alejandro G. Lopez. Data and statistical analysis: Irina Balan and Kai Xia. Data interpretation and intellectual contributions: Irina Balan, Alejandro G. Lopez, Thomas Gilmore, Michael Bremmer, Todd K. O'Buckley, Kai Xia, Christian S. Hendershot and A. Leslie Morrow. Manuscript draft: Irina Balan. Manuscript editing and approval: Irina Balan, Alejandro G. Lopez, Thomas Gilmore, Michael Bremmer, Todd K. O'Buckley, Kai Xia, Christian S. Hendershot and A. Leslie Morrow.

## Conflicts of Interest

The authors declare no conflicts of interest.

## Supporting information


**Table S1:** Group, sex, and interaction effects for each analyte, with effect sizes and confidence intervals.
**Table S2:** Linear regression analyses of biomarker levels predicting AUDIT and AUDIT‐C scores, with sensitivity analysis following MAD‐based outlier removal.
**Table S3:** Descriptive statistics of biomarker levels in participants with AUDIT ≥ 6 (High) and AUDIT < 6 (Low).
**Figure S1:** Diagnostic plots evaluating the assumptions of linear regression for IL‐1β in predicting AUDIT scores.
**Figure S2:** Diagnostic plots evaluating linear regression assumptions for the associations between IL‐1β, IL‐7, or CCL11 and AUDIT‐C scores.
**Figure S3:** Immune markers ranked by predictive importance in Random Forest models with 5‐fold cross‐validation (100 repetitions).
**Figure S4:** Associations between IL‐1β and AUDIT or AUDIT‐C scores, stratified by alcohol risk group.

## Data Availability

Data and the full R scripts are available upon reasonable request.
