# Supplementary material for: Identification of Interleukin‐1β in Whole Blood as a Candidate Biomarker for Alcohol Use Disorder Risk Based on AUDIT Scores
Source: Addict Biol. 2025 Sep 16;30(9):e70088. doi: 10.1111/adb.70088 (PMC12439176; doi:10.1111/adb.70088)
Supplement: Supplementary file 1 — Table S1: Group, sex, and interaction effects for each analyte, with effect sizes and confidence intervals. Table S2: Linear regression analyses of biomarker levels predicting AUDIT and AUDIT‐C scores, with sensitivity analysis following MAD‐based outlier removal. Table S3: Descriptive statistics of biomarker levels in participants with AUDIT ≥ 6 (High) and AUDIT < 6 (Low). Figure S1: Diagnostic plots evaluating the assumptions of linear regression for IL‐1β in predicting AUDIT scores. Figure S2: Diagnostic plots evaluating linear regression assumptions for the associations between IL‐1β, IL‐7, or CCL11 and AUDIT‐C scores. Figure S3: Immune markers ranked by predictive importance in Random Forest models with 5‐fold cross‐validation (100 repetitions). Figure S4: Associations between IL‐1β and AUDIT or AUDIT‐C scores, stratified by alcohol risk group. [file ADB-30-e70088-s001.pdf]

Supplementary Materials

Identification of IL-1β in Whole Blood as a Candidate Biomarker for Alcohol Use Disorder

Irina Balan, Alejandro G. Lopez, Thomas Gilmore, Michael Bremmer, Todd K. O'Buckley, Kai Xia, Christian S. Hendershot and A. Leslie Morrow

Materials and Methods

Blood cell lysate and supernatant preparation

Whole blood (~4 ml per participant) was collected into sodium heparin-coated Vacutainer® Plastic Tubes (BD, Cat. No. 367878). Immediately following collection, tubes were gently inverted 8–10 times to ensure anticoagulant mixing and prevent clotting, then placed on ice and transported to the laboratory within 15 minutes to minimize blood cell degradation.

Blood cell lysate and supernatant preparation were performed as previously described [1,2], using detergent-based lysis and sonication to extract both intracellular and extracellular biomarker fractions (including extracellular vesicles) from whole blood. Briefly, 1 ml of whole blood was aliquoted into each of three pre-labeled 2 ml microcentrifuge tubes designated for lysate preparation, generating three technical replicates per participant.

An equal volume (1 ml) of freshly prepared, ice-cold RIPA buffer (150 mM NaCl, 1.0% IGEPAL® CA-630, 0.5% sodium deoxycholate, 0.1% SDS, 50 mM Tris, pH 8.0; Sigma-Aldrich, Cat. No. R0278), supplemented with protease inhibitor cocktail (1:100; Sigma, Cat. No. P8340) and phosphatase inhibitor cocktail 3 (1:100; Sigma, Cat. No. P0044), was added to each tube. The samples were briefly vortexed and centrifuged at 9,500 × *g* for 5 minutes at 4 °C. The resulting supernatants, representing the extracellular biomarker-containing fraction and collected as three technical replicates per participant, were carefully transferred into labeled tubes, snap-frozen on dry ice, and stored at -80 °C until analysis.

The remaining cell pellets were gently drained of residual liquid and resuspended in 150 µl of ice-cold RIPA buffer supplemented with protease and phosphatase inhibitors. Samples were vortexed for 30 seconds and incubated on ice for 15 minutes. To disrupt cellular membranes and extracellular vesicles, and to prevent protein aggregation, samples were sonicated using a Qsonica Q500 ultrasonic processor (Qsonica, Newtown, CT, USA) fitted with a cup horn attachment (Model No. 431C2). Sonication was performed in a chilled water bath (1/3 ice, 2/3 cold water) at 80% amplitude for two 30-second cycles, with a 30-second cooling interval on ice between sonication bursts to prevent heat-induced protein degradation.

Samples were then centrifuged at 15,000 × g for 30 minutes at 4°C. The final supernatants, representing the intracellular biomarker-rich cell lysate fraction as well as the contents of extracellular vesicles, were transferred into new tubes, snap-frozen on dry ice, and stored at -80°C until Luminex assay analysis.

Luminex Multiplex Immunoassays

The assay was performed using the ProcartaPlex™ Multiplex Immunoassay Kit (Thermo Fisher Scientific, Catalog #PPX-14-MXCE7DR).

A key protocol modification involved the preincubation of RIPA-treated samples in Platinum Assay Buffer (Thermo Fisher, EPXP-11113-000) to reduce matrix interference from detergents and enhance antibody binding efficiency. Specifically, 50 µl of each sample (cell lysate or supernatant) was diluted 3-fold by adding 100 µL of Platinum Buffer, followed by 2-hour preincubation at 4°C with shaking at 600 rpm. Standards were also diluted 3-fold (50 µl of reconstituted standard + 100 µl Platinum Buffer) to match the sample matrix.

After preparation, 150 µl of each diluted sample (technical triplicates) and standard (duplicates) was transferred to wells of a black 96-well plate containing magnetic beads conjugated to analyte-specific capture antibodies. Plates were sealed, covered with black lids to minimize light exposure, and incubated on an orbital shaker at 600 rpm - first for 30 minutes at room temperature, followed by overnight incubation at 4 °C.

The next day, plates were brought to room temperature, washed twice with 1× Wash Buffer, and incubated with 25 µl of biotinylated detection antibody mix for 1 hour at room temperature while shaking at 600 rpm. After two additional washes, 50 µl of streptavidin–phycoerythrin (SA–PE) was added and incubated for 30 minutes, followed by a final wash step.

Prior to reading, 120 µl of 1× Wash Buffer was added to each well, and plates were shaken for 5 minutes at 600 rpm. Importantly, Wash Buffer (1x) was used instead of the standard Reading Buffer to maintain bead suspension and fluorescence signal integrity. Wash Buffer reduces background noise and prevents bead aggregation, which can interfere with fluorescence detection and compromise data quality, especially in detergent-containing samples.

Plates were read using a Luminex 200 system, and biomarker concentrations were calculated from standard curves generated using 4-fold serial dilutions of standards. Data were analyzed using a 5-parameter logistic (5-PL) regression model in the Thermo Fisher Procartaplex Analysis App. As both samples and standards were diluted identically, no dilution correction was applied during interpolation.

To express biomarker levels in pg/ml of whole blood, the following correction factors were applied:

1. For cell lysates, the pellet from 1 ml of whole blood was resuspended in 150 µl of RIPA buffer. Thus, 50 µl of lysate corresponds to 333.3 µl of blood, and measured concentrations were multiplied by 0.15 to reflect the original volume.
2. For supernatants, which were generated by a 2-fold dilution with RIPA buffer, measured concentrations were multiplied by 2 to correct for the dilution factor.

Final biomarker concentrations are reported as pg/ml of whole blood, representing the combined contributions from both cell lysate + supernatant of whole blood preparations.

Statistical Analysis

All statistical analyses were conducted in R (version 4.4.3) within the RStudio environment (version 2024.12.1).

Kruskal-Wallis Test

R Packages and Functions:

stats: shapiro.test() (normality), kruskal.test() (comparisons), p.adjust() (FDR correction)
dplyr: filter(), mutate(), group_by(), %>% (data manipulation)
ggplot2: ggplot(), geom_boxplot(), geom_jitter() (visualization)
ggpubr: stat_compare_means() (p-value annotations)

reshape2: melt() (data reshaping)
tidyverse: Integrated package suite for data wrangling and reproducibility

Linear Regression
R Packages and Functions:

readxl: read_excel() (import data)
dplyr: select() (column selection), filter() (outlier removal), na.omit() (handle missing data), %>% (streamline operations)
stats: lm() (linear model fitting), summary() (model statistics), coef() (extract coefficients and p-values), mad() (median absolute deviation), median(), p.adjust(method="fdr") (FDR correction)
ggplot2: ggplot(), aes(), geom_point(), geom_smooth() (regression line), labs() (axis labels), theme_minimal() (style), ggsave() (export plots)
openxlsx: createWorkbook(), addWorksheet(), writeData(), saveWorkbook() (Excel export)

Random Forest Model
R Packages and Functions:

randomForest: randomForest() (model fitting), importance() (variable importance)
dplyr: Variable selection and data manipulation prior to analysis
stats: cor(), mean(), sd() (aggregation of results across repetitions)
ggplot2: ggplot(), geom_col(), ggsave() (feature importance plots)
openxlsx: createWorkbook(), writeData(), saveWorkbook() (export to Excel)

5-Fold Cross-Validated Random Forest Model
R Packages and Functions:

randomForest: randomForest() (model fitting), importance() (variable importance scores: MDA and MDG)
caret: createFolds() (generation of stratified 5-fold cross-validation splits)
dplyr: %>%, na.omit(), arrange() (data wrangling and output formatting)
stats: cor(), mean(), sd() (performance metric computation: R^2, RMSE)
openxlsx: createWorkbook(), addWorksheet(), writeData(), saveWorkbook() (Excel export of variable importance and model performance)
ggplot2: ggplot(), geom_col(), coord_flip(), labs(), theme_minimal() (visualization of biomarker importance rankings)

Moderated Multiple Regression (MMR)
R Packages and Functions

readxl: Used to read Excel files containing the biomarker and questionnaire data. (read_excel())
dplyr: Used for data manipulation, including selecting columns, removing missing values (na.omit()), and centering variables. Functions used include filter(), mutate(), scale(), and the pipe operator %>%.

stats (base R): Used for statistical modeling. The lm() function was used to fit linear models, summary() provided model statistics, and coef() was used to extract regression coefficients and p-values.
p.adjust(): Base R function used with method = "fdr" to adjust interaction p-values for multiple comparisons using the Benjamini–Hochberg method.
openxlsx: Used to export results into Excel format. Key functions included createWorkbook(), addWorksheet(), writeData(), and saveWorkbook().

Receiver Operating Characteristic (ROC) Analysis
R Packages and Functions – ROC Analysis
readxl: Used to import Excel files containing participant data.
dplyr: Used for data selection, filtering, and handling missing values (select(), na.omit()).
pROC: Core package used for ROC analysis. roc() generates the ROC curve; coords() extracts sensitivity, specificity, and thresholds; auc() computes the area under the curve.
ggplot2: Used to plot ROC curves. geom_line() creates the ROC curve; geom_point() marks the optimal threshold; annotate() adds threshold labels; ggsave() exports plots as PNG images.
writexl: Used to export the summary statistics to Excel using write_xlsx().

Principal Component Analysis (PCA)
R Packages and Functions
readxl: Used to import Excel data containing biomarker values. read_excel(): loads the dataset
factoextra: Used to perform PCA and visualize variable contributions. prcomp(): performs PCA. fviz_pca_var(): creates biplots of variables colored by $\cos^2$ contribution.
ggplot2: Used to plot bar charts of top contributors. ggplot(), geom_bar(), coord_flip(), labs(), theme_minimal(), ggsave(): generate and save top contributor plots.
openxlsx: Used to export top contributors to Excel. createWorkbook(), addWorksheet(), writeData(), saveWorkbook(): export ranked contributors for PC1 and PC2.

Linear Regression Diagnostics
R Packages and Functions:
readxl: read_excel() (load data from Excel spreadsheet)
stats (base R):
lm() (fit linear regression model)
fitted() (extract predicted values from the model)
residuals() (extract model residuals)
qqnorm(), qqline() (create Q–Q plot to assess normality of residuals)
plot(), abline() (generate residuals vs. fitted values plot)
par() (configure multi-panel plotting layout)

Results Tables:

Table S1. Group, sex, and interaction effects for each analyte, with effect sizes and confidence intervals.

Analyte	High vs Low Group /H	High vs Low Group/df	High vs Low Group /p	High vs Low Group /adj p	Sex/ H	Sex /df	Sex/p	Sex/adj p	Inter/ H	Inter/ df	Inter/ p	Inter/ adj p	Cohen d	Cohen CI lower	Cohen CI upper
**BDNF**	1.1	1	0.29	0.41	2.89	1	0.09	0.59	6.0	3	0.11	0.26	0.22	-0.54	0.98
**CCL11**	7.1	1	0.008	**0.03**	0.76	1	0.38	0.77	8.3	3	0.04	0.11	**1.10**	0.29	1.91
**HMGB1**	0.9	1	0.33	0.43	0.03	1	0.85	0.96	1.0	3	0.80	0.94	-0.41	-1.17	0.36
**IL-1β**	7.9	1	0.005	**0.03**	0.02	1	0.89	0.96	9.7	3	0.02	0.09	**0.97**	0.18	1.77
**IL-3**	0.1	1	0.78	0.84	0.26	1	0.61	0.90	0.3	3	0.95	0.95	0.19	-0.57	0.95
**IL-5**	3.1	1	0.08	0.19	0.30	1	0.58	0.90	4.0	3	0.26	0.46	0.56	-0.21	1.32
**IL-6**	1.3	1	0.25	0.39	0.00	1	0.96	0.96	2.1	3	0.56	0.78	0.63	-0.14	1.40
**IL-7**	7.6	1	0.006	**0.03**	1.90	1	0.17	0.59	9.5	3	0.02	0.09	**1.17**	0.36	1.99
**IL-8**	3.2	1	0.07	0.19	5.28	1	0.02	0.30	11.2	3	0.01	0.09	0.46	-0.30	1.23
**IL-17A**	0.00	1	0.95	0.95	0.84	1	0.36	0.77	0.9	3	0.83	0.94	0.50	-0.26	1.27
**IL-18**	9.2	1	0.002	**0.03**	0.08	1	0.78	0.96	9.3	3	0.03	0.09	**1.25**	0.42	2.07
**MCP-1**	0.4	1	0.55	0.64	0.21	1	0.65	0.90	0.7	3	0.87	0.94	0.38	-0.38	1.14
**MIP-1β**	2.6	1	0.11	0.22	1.02	1	0.31	0.77	3.6	3	0.31	0.48	0.73	-0.05	1.51
**TNF-α**	1.3	1	0.25	0.39	2.30	1	0.13	0.59	4.0	3	0.26	0.46	0.50	-0.27	1.26

This table presents the results of Kruskal-Wallis tests examining the main effects of group (high-risk: AUDIT ≥ 6 vs. low-risk: AUDIT < 6), sex (male vs. female), and their interaction (Group × Sex) on analyte levels in a sample of 28 participants (14 per group, with 7 males and 7 females in each). Only false discovery rate (FDR)-adjusted p-values (adj p) are reported to assess statistical significance. "Inter" refers to the group-by-sex interaction term.

Significant group differences (adj p < 0.05) were observed for CCL11, IL-1β, IL-7, and IL-18. No significant main effects of sex or Group × Sex interaction effects were found after adjustment. However, these exploratory analyses of Group × Sex interactions were underpowered due to the modest sample size and should be interpreted cautiously.

Effect sizes (Cohen's d), with 95% confidence intervals (CI), were calculated to estimate the magnitude of group differences. Among biomarkers with significant group effects, large effect sizes further support the robustness of the observed differences between the high-risk and low-risk groups.

Bolded FDR-adjusted p-values indicate statistical significance (adj p < 0.05); bolded Cohen's d values denote large effect sizes.

Table S2. Linear regression analyses of biomarker levels predicting AUDIT and AUDIT-C scores, with sensitivity analysis following MAD-based outlier removal

Biomarker	AUDIT vs. Biomarker (Predictor)						AUDIT-C vs. Biomarker (Predictor)					
	Full Dataset			Sensitivity Analysis			Full Dataset			Sensitivity Analysis		
	R^2	p	adj p	R^2	p	adj p	R^2	p	adj p	R^2	p	adj p
**BDNF**	0.03	0.37	0.43	0.09	0.12	0.19	0.08	0.15	0.21	0.14	0.05	0.09
**CCL11**	0.14	0.05	0.12	0.14	0.05	0.14	0.25	0.006	**0.03**	0.25	0.006	**0.03**
**HMGB1**	0.01	0.59	0.62	0.01	0.59	0.69	0.04	0.30	0.30	0.04	0.30	0.42
**IL-1β**	0.51	0.00002	**0.0002**	0.34	0.002	**0.02**	0.38	0.0005	**0.007**	0.30	0.004	**0.03**
**IL-3**	0.10	0.11	0.18	0.04	0.34	0.48	0.06	0.22	0.25	0.02	0.51	0.59
**IL-5**	0.01	0.62	0.62	0.003	0.80	0.81	0.05	0.23	0.25	0.00	0.92	0.92
**IL-6**	0.23	0.01	0.06	0.23	0.01	0.05	0.17	0.03	0.08	0.17	0.03	0.08
**IL-7**	0.14	0.046	0.12	0.16	0.04	0.13	0.33	0.001	**0.009**	0.30	0.003	**0.03**
**IL-8**	0.22	0.01	0.06	0.23	0.01	0.05	0.13	0.06	0.10	0.14	0.05	0.09
**IL-17A**	0.09	0.13	0.18	0.002	0.81	0.81	0.12	0.08	0.12	0.01	0.62	0.66
**IL-18**	0.09	0.12	0.18	0.09	0.12	0.19	0.16	0.03	0.08	0.16	0.03	0.08
**MCP-1**	0.06	0.22	0.28	0.01	0.59	0.69	0.05	0.23	0.25	0.03	0.39	0.50
**MIP-1β**	0.11	0.08	0.16	0.11	0.08	0.18	0.17	0.03	0.08	0.17	0.03	0.08
**TNF-α**	0.18	0.03	0.09	0.10	0.12	0.19	0.15	0.04	0.08	0.07	0.20	0.32

This table presents the results of linear regression analyses assessing associations between individual biomarker levels and continuous AUDIT and AUDIT-C scores in 28 participants. For each biomarker, two models were constructed: one using the full dataset and another excluding outliers identified as values exceeding 6.5 times the median absolute deviation (MAD), a robust method for detecting extreme deviations from central tendency. This approach, known as sensitivity analysis, evaluates the robustness of the associations by determining whether the results hold true when potential outliers are removed. Each model reports the coefficient of determination (R^2), raw p-value, and false discovery rate (FDR)–adjusted p-value (adj p). Significant associations (adj $p < 0.05$) were observed between IL-1β levels and both AUDIT and AUDIT-C scores across both models. Additionally, CCL11 and IL-7 levels were significantly associated with AUDIT-C scores in both the full dataset and the sensitivity analysis. No other biomarkers demonstrated significant associations after FDR correction. In the table, FDR-adjusted p-values less than 0.05 are indicated in bold to denote statistical significance after correction for multiple comparisons.

Table S3. Descriptive statistics of biomarker levels in participants with AUDIT ≥ 6 (High) and AUDIT < 6 (Low).

AUDIT	Biomarker	Mean (pg/ml)	SD (pg/ml)	CV %
High	**BDNF**	1623.1	1036.1	63.8
	**CCL11**	97.9	58.9	60.2
	**HMGB1**	194217.0	100800.5	51.9
	**IL-1β**	8.6	8.9	103.8
	**IL-3**	38.0	80.1	210.5
	**IL-5**	8.0	12.5	156.5
	**IL-6**	4.8	3.7	78.6
	**IL-7**	2.6	1.6	61.5
	**IL-8**	11.0	26.8	242.8
	**IL-17A**	2.3	4.3	182.8
	**IL-18**	637.2	347.1	54.5
	**MCP-1**	1388.7	982.7	70.8
	**MIP-1β**	61.5	40.7	66.1
	**TNF-α**	11.0	25.0	228.2
Low	**BDNF**	1355.6	1302.6	96.1
	**CCL11**	45.3	28.6	63.2
	**HMGB1**	235186.0	94882.6	40.3
	**IL-1β**	2.1	1.8	83.5
	**IL-3**	26.1	31.2	119.3
	**IL-5**	2.7	3.6	130.6
	**IL-6**	2.9	1.7	59.7
	**IL-7**	1.0	0.9	84.9
	**IL-8**	2.0	1.9	92.7
	**IL-17A**	0.7	0.7	101.2
	**IL-18**	293.2	150.9	51.5
	**MCP-1**	1063.4	638.8	60.1
	**MIP-1β**	37.3	20.5	55.0
	**TNF-α**	1.9	2.3	123.5

Mean biomarker levels (pg/ml), standard deviations (SD), and coefficients of variation (CV%) are reported for participants with AUDIT scores ≥ 6 and < 6. These groups are referred to as high-risk (High; AUDIT ≥ 6) and low-risk (Low; AUDIT < 6) groups, each consisting of 14 participants (7 males and 7 females). The coefficient of variation (CV), expressed as a percentage, reflects relative variability in biomarker levels across individuals within each group.

Several biomarkers in the AUDIT ≥ 6 group, including TNF-α, IL-8, IL-3, and IL-17A, showed notably high CVs, suggesting elevated inter-individual variability in inflammatory responses compared to the AUDIT < 6 group.

Figures:

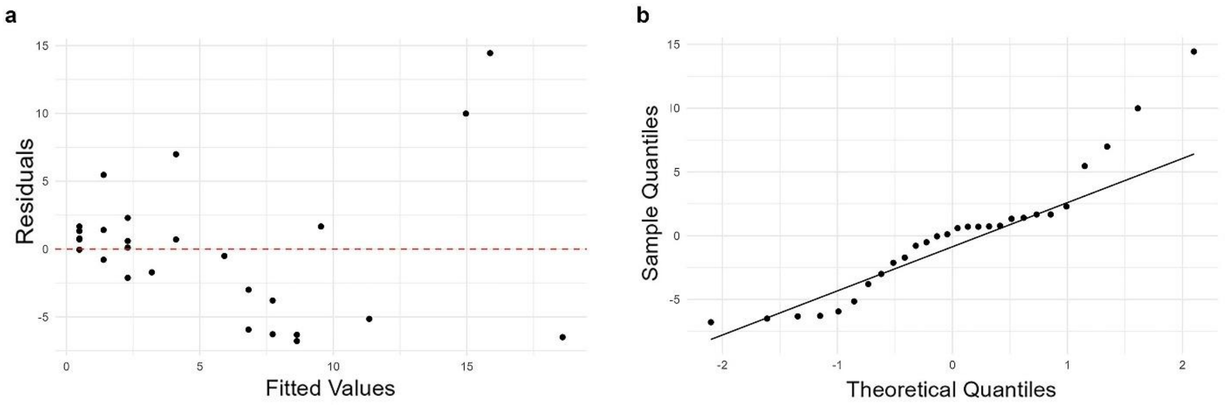

Figure S1. Diagnostic plots evaluating the assumptions of linear regression for IL-1β in predicting AUDIT scores.
(a) Residuals vs. Fitted Values Plot: This plot displays the residuals (the differences between observed and predicted values) against the fitted values from the model. The residuals are scattered approximately evenly around zero without a clear pattern, indicating no substantial non-linearity or heteroscedasticity (non-constant variance).
(b) Q–Q (Quantile–Quantile) Plot: This plot assesses whether the residuals follow a normal distribution by comparing their quantiles to those of a theoretical normal distribution. The points mostly follow the diagonal reference line, with only minor deviations at the tails, supporting the assumption of approximate normality.
Together, these plots support the validity of the linear regression model used to evaluate the relationship between IL-1β and AUDIT scores.

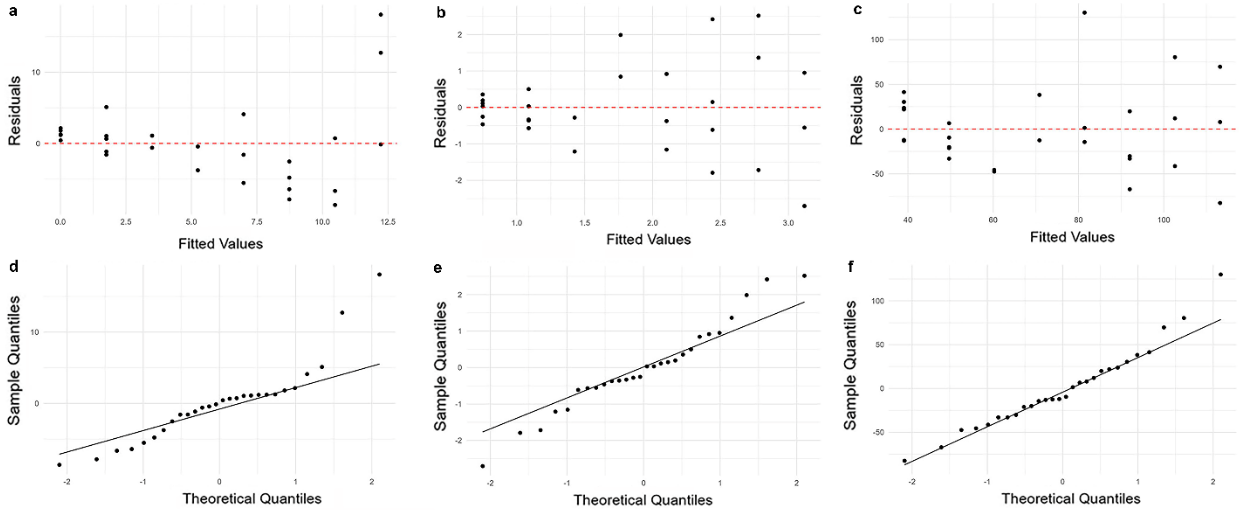

Figure S2. Diagnostic plots evaluating linear regression assumptions for the associations between IL-1β, IL-7, or CCL11 and AUDIT-C scores.
(a, d) IL-1β; (b, e) IL-7; (c, f) CCL11.
Top Panels (a–c): Residuals vs. Fitted Values Plots
These plots assess whether residuals are randomly distributed across fitted values, which supports the assumptions of linearity and homoscedasticity (constant variance). For all three models, the residuals appear reasonably scattered around the horizontal reference line at zero, with no major patterns or funnel shapes observed. This suggests no substantial non-linearity or heteroscedasticity.
Bottom Panels (d–f): Q–Q (Quantile–Quantile) Plots
These plots compare the distribution of residuals to a theoretical normal distribution. If residuals are normally distributed, the points should fall along the diagonal reference line. All three models show approximate alignment with the line, with only minor deviations observed at the extremes. This indicates acceptable adherence to the normality assumption.
Together, these diagnostic plots support the overall validity of the linear regression models for IL-1β, IL-7, and CCL11 in predicting AUDIT-C scores.

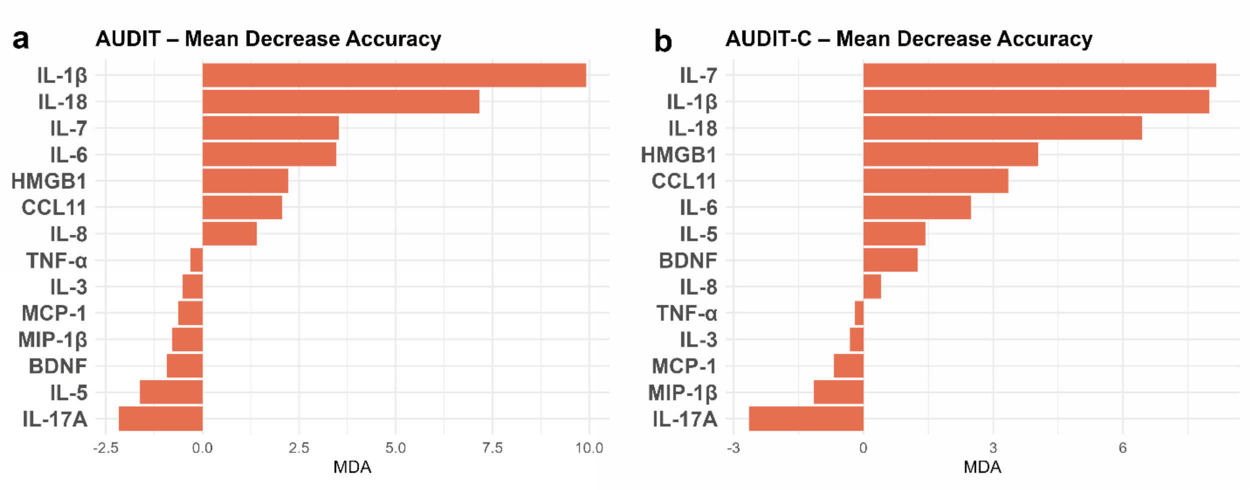

Figure S3. Immune markers ranked by predictive importance in Random Forest models with 5-fold cross-validation (100 repetitions).

(a) For AUDIT scores, the model achieved a mean R^2 of 0.50 and RMSE of 4.5 (SD = 0 across repetitions). IL-1β remained the top predictor (MDA = 9.9; MDG = 125.5), followed by IL-18, IL-7, IL-6, HMGB1, CCL11, and IL-8.

(b) For AUDIT-C scores, the model yielded a mean R^2 of 0.46 and RMSE of 2.1 (SD = 0). IL-1β and IL-7 showed nearly identical importance (IL-1β: MDA = 8.0, MDG = 16.9; IL-7: MDA = 8.2, MDG = 14.5), followed by IL-18, HMGB1, CCL11, and IL-6. IL-5 and BDNF also contributed modestly. Other markers, including IL-8, TNF-α, IL-3, MCP-1, MIP-1β, and IL-17A, exhibited minimal or negative importance. Results highlight the consistent predictive strength of IL-1β, along with other immune mediators, in explaining variance in alcohol-related risk scores.

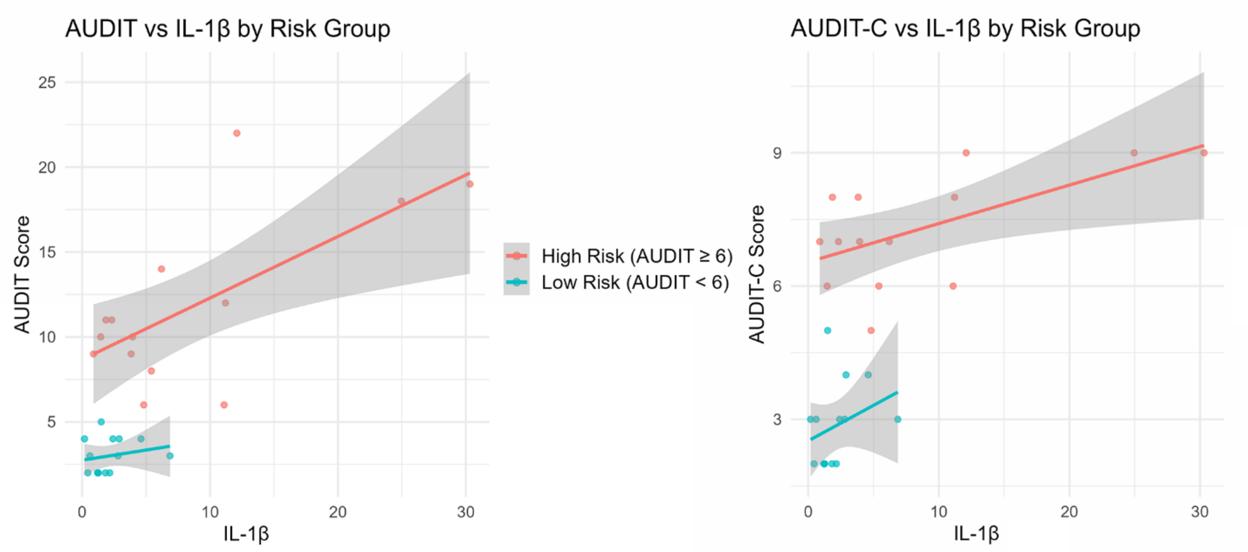

Figure S4. Associations between IL-1β and AUDIT or AUDIT-C scores, stratified by alcohol risk group.
Linear regression models were fit separately for low-risk (AUDIT < 6; blue) and high-risk (AUDIT ≥ 6; red) participants. Left panel: IL-1β was significantly associated with AUDIT scores in the high-risk group ($R^2 = 0.45$, $p = 0.009$, FDR-adjusted $p = 0.045$), but not in the low-risk group ($R^2 = 0.04$, $p = 0.48$). Right panel: IL-1β was significantly associated with AUDIT-C scores in the high-risk group ($R^2 = 0.37$, $p = 0.02$, FDR-adjusted $p = 0.06$), but not in the low-risk group ($R^2 = 0.09$, $p = 0.30$). Shaded bands represent 95% confidence intervals. Results indicate that IL-1β levels are selectively associated with alcohol use severity in individuals at higher risk.

References

1 Balan I, Patterson R, Boero G, Krohn H, O'Buckley TK, Meltzer-Brody S, et al. Brexanolone therapeutics in post-partum depression involves inhibition of systemic inflammatory pathways. EBioMedicine. 2023;89:104473.

2 Balan I, Lopez AG, Morrow AL. Multiplex Immunoassay for Biomarker Profiling of Whole Blood Cell Lysates and Supernatants and Pathogen Response in Neat Whole Blood Cultures. Methods and Protocols. 2025;8(3):46.
